# Is adherence to the 24-hour movement guidelines associated with a reduced risk of adiposity among children and adolescents?

**DOI:** 10.1186/s12889-020-09213-3

**Published:** 2020-07-16

**Authors:** Lukáš Jakubec, Aleš Gába, Jan Dygrýn, Lukáš Rubín, Adam Šimůnek, Erik Sigmund

**Affiliations:** 1 Faculty of Physical Culture, Palacký University Olomouc, třída Míru 117, 779 00 Olomouc, Czech Republic; 2 Faculty of Science, Humanities and Education, Technical University of Liberec, Liberec, Czech Republic

**Keywords:** Accelerometry, Childhood obesity, Physical activity, Screen time, Sleep

## Abstract

**Background:**

Little is known about the combined effect of physical activity (PA), recreational screen time (ST), and sleep in preventing childhood obesity. Hence, this study aimed to analyze the associations between meeting the PA, ST, and sleep recommendations within the 24-hour movement guidelines and adiposity indicators among children and adolescents.

**Methods:**

A total of 679 children and adolescents aged 8–18 years were included. The time spent in moderate-to-vigorous PA and the sleep duration were estimated from raw data from a wrist-worn accelerometer. Recreational ST was reported by the child or parent. Body mass index (BMI) *z*-score, fat mass percentage (FM%), and visceral adipose tissue (VAT) were used as adiposity indicators. Participants with ≥ 60 min/day of moderate-to-vigorous PA, < 2 h/day of recreational ST, and uninterrupted sleep for 9–11 h/day (for children) or 8–10 h/day (for adolescents) were considered to meet the overall 24-hour movement guidelines.

**Results:**

Meeting the ST only recommendation was associated with reduced odds of a high BMI *z*-score (odds ratio [OR] = 0.38, 95% confidence interval [CI]: 0.17–0.89), excess FM% (OR = 0.34, 95% CI: 0.13–0.93), and excess VAT (OR = 0.27, 95% CI: 0.10–0.74) in adolescents. Significantly reduced odds of a high BMI *z*-score was associated with meeting the combination of the ST and sleep recommendations (OR = 0.11, 95% CI: 0.01–0.89). Adolescents who met one recommendation (OR = 0.51, 95% CI: 0.27–0.96) or any two recommendations (OR = 0.33, 95% CI: 0.11–0.94) had reduced ORs of having a high BMI *z*-score. Adolescents had lower odds of having excess VAT if they met one recommendation (OR = 0.39, 95% CI: 0.19–0.81) or any two recommendations (OR = 0.25, 95% CI: 0.07–0.90). No significant associations were found in children.

**Conclusions:**

The present study showed no associations between meeting all three recommendations within the 24-hour movement guidelines and adiposity indicators. However, meeting ST only recommendation and the combination of the ST and sleep recommendations was associated with a reduced risk of excess adiposity. This finding should be considered when designing effective strategies and interventions to prevent childhood obesity.

## Background

Obesity has become a major health challenge worldwide because its prevalence has substantially increased in the past four decades [1, 2]. Childhood obesity is significantly associated with an increased risk of incident severe obesity in adulthood [3, 4], as well as several non-communicable diseases and all-cause mortality later in life [5]. Moreover, the severity of health consequences associated with obesity might be influenced by body fat distribution, especially with increasing visceral adipose tissue (VAT) levels [6]. Obesity also has a high economic burden. Member countries of the Organisation for Economic Co-operation and Development spend 331 billion US dollars annually to treat diseases caused by overweight and obesity, which is equal to 8.4% of the total health-care costs [7]. Thus, there is an urgent need to prevent overweight and obesity to save societal resources [8].

In the past decades, several global and national intervention strategies were developed to prevent the increasing levels of overweight and obesity in childhood. These strategies mainly focused on optimizing energy balance by changing the level of physical activity (PA) and/or by improving dietary patterns [9]. A previous study has shown that single-component interventions, including PA, may have an weak positive effect on weight loss [10]. This result may be attributed to the fact that interventions exclusively focused on moderate-to-vigorous physical activity (MVPA), which represents < 5% of the time in a 24-h period [11], while ignoring other movement behaviors. Excessive sedentary behavior (SB) and short sleep duration are also well-known contributors to the obesity risk [12, 13]. For this reason, all movement behaviors within the 24-h period should be considered for designing effective interventions to prevent childhood obesity.

On the basis of the understanding of the effects of human movement behaviors during the entire 24-h period on health [11], the first 24-hour movement guidelines for children and adolescents were developed and presented in Canada [14] in 2016. After 1 year, the guidelines for children aged < 5 years were released [15]. Thereafter, Australia, New Zealand, South Africa, and the World Health Organization introduced the 24-hour movement guidelines for children in different age groups [16–20]. Unlike the previous guidelines, which mainly focused on the time spent in MVPA, the 24-hour movement guidelines incorporate sleep and recreational screen time (ST) recommendations.

The 24-hour movement guidelines were established to encourage children and adolescents to achieve optimal balance between different behaviors within a day and to prevent them from developing various non-communicable diseases including obesity. To date, only a few studies have analyzed the associations between meeting the 24-hour movement guidelines and adiposity indicators in the pediatric population [21, 22]. The main limitations of these studies were that all recommendations within the 24-hour movement guidelines were obtained from self-report questionnaires [21] and that a narrow age group was investigated [22]. Hence, this cross-sectional study used device-measured PA and sleep, and self- or parental-reported recreational ST among children and adolescents to (1) examine the associations between adherence to the 24-hour movement guidelines and adiposity indicators, and (2) identify whether meeting different numbers and specific combinations within the 24-hour movement guidelines is associated with the adiposity status.

## Methods

### Participants

The study included children (age 8–13 years) and adolescents (age 14–18 years) who were recruited from seven elementary schools and four high schools in the Czech Republic. The parents or guardians of the participants received information leaflets about the complete description of the study after the school management approved the research. The main inclusion criteria were participant age and good health condition, which was reported by their parents. Those participants whose parents reported medical complications that could affect PA and sleep were excluded from study. The sample comprised 907 children and adolescents who agreed to participate. However, approximately 25% of the participants were excluded because they voluntarily withdrew from the study or became ill (*n* = 45), provided incomplete data (*n* = 129), had data that could not be assessed because of technical failures (*n* = 17), or did not meet the criteria for accelerometer wear time (*n* = 37). Therefore, the final sample comprised 679 participants (57% girls) who provided complete data. The characteristics of the participants are shown in Table 1.

**Table 1:** Descriptive characteristics and weight status of the study sample

	Total ***N*** = 679		Children ***n*** = 355		Adolescents ***n*** = 324		***p***-value^c^
	Mean	SD	Mean	SD	Mean	SD	
Age (years)	13.9	2.8	11.7	1.6	16.3	1.3	< 0.001
Height (cm)	160.5	14.1	151.6	12.0	170.2	8.8	< 0.001
Weight (kg)	52.9	15.0	43.6	11.3	63.0	11.6	< 0.001
BMI *z*-score	0.22	1.06	0.24	1.13	0.20	0.99	0.587
Fat mass (%)	20.0	8.7	19.3	8.3	20.7	9.1	0.035
Visceral adipose tissue (cm^2^)	48.5	31.1	42.7	28.1	54.9	32.9	< 0.001
**Weight status**^a^ (% of *n*)							
Underweight	1.3		2.0		0.6		0.112
Normal weight	76.3		73.5		79.3		0.076
Overweight	17.1		17.2		17.0		0.945
Obese	5.3		7.3		3.1		0.015
MVPA (min/day)^b^	49.1	23.9	58.1	24.3	39.3	19.1	< 0.001
ST (h/day)	3.0	2.0	3.0	1.8	2.8	2.1	0.206
SL (h/day)^b^	8.1	0.9	8.6	0.7	7.5	0.8	< 0.001

BMI – body mass index, MVPA – moderate-to-vigorous physical activity, ST – screen time, SL – sleep

^a^Based on the BMI *z*-score

^b^Adjusted to 24 h

^c^The differences between age categories were analyzed with the independent *t*-test for continuous variables and the chi-square test for categorical variables

### Procedure

Data collection was conducted during the spring and fall seasons between March 2018 and April 2019. Monitoring was performed during weeks with a regular school schedule. The participants in each school were visited twice. During the first visit, accelerometers, daily logs, and questionnaires were distributed. The participants were instructed on how to wear the accelerometer and how to properly enter data in the daily log. Assessment of adiposity was also performed. Accelerometer monitoring was started in the midnight after the initial visit. Thus, the participants were instructed to wear the device before bedtime. The second visit was conducted 8 days after the initial visit, and the accelerometers, daily logs, and filled questionnaires were collected.

### Physical activity and sleep

PA and sleep were measured using ActiGraph tri-axial accelerometers (ActiGraph, Pensacola, FL, USA), including wGT3X-BT and GT9X Link for children and adolescents, respectively. Both scientific-grade accelerometers are commonly used in the PA [23–25] and sleep [26, 27] research and provide reliable and valid measures [28–30]. The participants wore the accelerometer on the non-dominant wrist for 24 h/day for 7 consecutive days, except when bathing, swimming, and performing personal hygiene. These non-wear windows were recorded into the provided daily log together with the participants’ wake-up and fall-asleep time. In participants < 12 years old (i.e., those in the first stage of elementary school), assistance from the parent or teacher was needed to obtain the precise time when filling the daily log.

The accelerometers were initialized to collect acceleration data on three axes at a sampling rate of 100 Hz and were downloaded using ActiLife software version 6.13.3 (ActiGraph Corp., Pensacola, FL, USA). Data were saved as GT3X files before they were converted to the raw .csv file format. The open-source R package GGIR version 1.10–7 (https://cran.r-project.org/web/packages/GGIR/) was used to calculate the Euclidean Norm Minus One metric, with negative values rounded up to zero [31]. The time spent in MVPA was calculated without bout criteria using the > 201 mg threshold proposed by Hildebrand et al. [32]. The heuristic algorithm [33] guided by individual wake-up and fall-asleep times from the daily log was used to identify the sleep time. The day cycle was determined using the “from waking up to waking up the next day” approach, which means that a full night of sleep is included in a day cycle and that the duration of a day cycle may vary. Participants were excluded if they had fewer than three weekdays and one weekend day of valid wear (defined as ≥ 16 h per day), or if wear data for each 15-min period in the 24-h cycle were not available [34].

### Screen time

Recreational ST on weekdays and weekend days was obtained using the following questions from the Health Behaviour in School-aged Children study [35]: “About how many hours a day do you usually spend watching television, DVDs, videos (including YouTube or similar online service) in your free time on weekdays (weekend days)?” and “About how many hours a day do you usually spend playing games on a computer, games console (PlayStation, Xbox, etc.), smartphone, tablet, or similar electronic device in your free time on weekdays (weekend days)?” Nine response options were provided for each question (0, 0.5, 1, 2, 3, 4, 5, 6, and ≥ 7 h/day). The validity and reliability of the questions for the past 7 days has been proved in comparison with the 7-day 24-h diaries both on weekdays and at weekends [36].

A parent proxy report was required in children aged ≤12 years. In total, 16 (3%) participants had missing data for one or two ST items. The expectation-maximization algorithm was used to impute missing data. Those with missing items (three or all) were excluded from the analysis. The total ST was calculated by obtaining the sum of the weighted averages of ST during weekdays and weekends (weighted average = [average of weekdays ∙ 5 + average of weekend days ∙ 2] / 7).

### Adherence to the 24-hour movement guidelines

Participants with ≥ 60 min/day of MVPA, < 2 h/day of recreational ST, and uninterrupted sleep for 9–11 h/day (for children) or 8–10 h/day (for adolescents) were considered to meet the overall 24-hour movement guidelines [14].

### Adiposity indicators

Body mass index (BMI) *z*-score, fat mass percentage (FM%), and VAT were used as the indicators of adiposity. To calculate BMI, the height and weight of the participants were measured using a standard procedure with an accuracy of 0.1 cm and 0.1 kg, respectively. Sex- and age-specific BMI cutoff points were used to identify participants with overweight and obesity (i.e., BMI *z*-score > 1 standard deviation [SD]).

The total and visceral adiposity levels were measured using the InBody 720 device (InBody Co., Seoul, Korea), which is a multi-frequency bioimpedance analyzer used to measure resistance in broadband frequencies (1–1000 kHz) and reactance in mean frequencies (5–250 kHz). Bioimpedance analysis is considered suitable for assessing adiposity in a target population, as its validity has been confirmed [37]. The participants were instructed in advance to maintain proper hydration, avoid any vigorous exercise for at least 1 day before the examination, and fast at least 4 h before the examination. Assessment of adiposity was performed on school premises during school hours (8:00–13:30). Excess adiposity was defined as the sex- and age-specific 85th percentile of FM% and VAT.

### Potential confounding variables

We selected possible confounding factors that were available in the dataset and have been shown to be associated with adiposity indicators. Maternal obesity was based on BMI calculated from self-reported height and weight. Unhealthy diet was characterized by a high frequency of fast-food consumption (i.e., ≥ 1 portion per a week), and data about this diet were obtained using a single-item question. Maternal obesity and unhealthy diet have been dichotomized before analysis. Birth weight and maternal height and weight were reported by the parents. The parent-reported data of unhealthy diet were required only in children < 12 years old (i.e., those in the first stage of elementary school). The school location was also considered as the participants were recruited within clusters. However, the school location was not included in the final models as this potential confounder was not associated with the outcome variable and its omission did not reduce the overall significance of the regression models.

### Statistical analysis

All statistical analyses were conducted using the Statistical Package for the Social Sciences software version 25 (IBM Company, Armonk, NY, USA). The descriptive statistics of the outcome measures are presented as mean and SD. The differences between age groups were analyzed with the independent *t*-test for continuous variables and with the chi-square test for categorical variables.

A binary logistic regression analysis was performed to estimate the odds of excess adiposity in participants who met different numbers and combinations of recommendations within the 24-hour movement guidelines. Analysis was performed using two separate regression models. In the first model, odds ratios (ORs) with 95% confidence intervals (CIs) of having excess adiposity were calculated for those who met one, any two, or all three recommendations. In the second model, the ORs for all possible combinations within the 24-hour movement guidelines (i.e., eight categories) were calculated. Participants who did not meet any recommendation were used as the reference group in all analyses. The estimates were based on logistic regression models adjusted for sex, age, and confounding factors. The level of significance was set at *p* < 0.05.

## Results

The characteristics of the participants are shown in Table 1. The mean age of the total sample was 13.9 ± 2.8 years, and 22.4% of the participants were overweight and obese. The FM% and VAT of adolescents were significantly higher than those of children, by 1.4% (*p* = 0.035) and 12.2 cm^2^ (*p* < 0.001), respectively. Although the prevalence of overweight was almost equal between age groups, the prevalence of obesity was more than two-fold lower in adolescents than in children (*p* = 0.015). The MVPA and ST of adolescents were lower by 18.8 min/day (*p* < 0.001) and 1.1 h/day (*p* < 0.001), respectively, than those of the children. None of the participants had longer sleep duration as it is recommended.

Only 6.5% of children (Table 2) and 2.2% of adolescents (Table 3) met all 3 recommendations. Adolescents who met only one recommendation had lower odds of having a high BMI *z*-score (OR = 0.51, 95% CI: 0.27–0.96) and excess VAT (OR = 0.39, 95% CI: 0.19–0.81) than those who did not meet any recommendation. Furthermore, meeting any two recommendations was associated with lower odds of having a high BMI *z*-score (OR = 0.33, 95% CI: 0.11–0.94) and excess VAT (OR = 0.25, 95% CI: 0.07–0.90).

**Table 2:** Odds of excess adiposity in children who met different numbers and specific combinations of recommendations within the 24-hour movement guidelines (*n* = 355)

	Adherence	BMI ***z***-score > 1 SD			>85th percentile of FM%			>85th percentile of VAT		
	%	OR	(95% CI)	***p***-value	OR	(95% CI)	***p***-value	OR	(95% CI)	***p***-value
**Number of recommendations**										
None	33.0	Ref.			Ref.			Ref.		
One	37.7	1.44	(0.79–2.61)	0.234	1.05	(0.53–2.10)	0.891	1.05	(0.52–2.11)	0.898
Two	22.8	1.05	(0.51–2.15)	0.901	0.52	(0.20–1.33)	0.171	0.46	(0.17–1.23)	0.121
Three	6.5	0.33	(0.07–1.54)	0.158	0.25	(0.31–1.93)	0.187	0.26	(0.03–2.05)	0.199
**Combinations of recommendations**										
Did not meet any recommendation	33.0	Ref.			Ref.			Ref.		
PA only	18.3	1.66	(0.81–3.39)	0.165	0.94	(0.40–2.24)	0.889	1.09	(0.46–2.57)	0.849
ST only	11.0	1.06	(0.43–2.62)	0.907	1.08	(0.40–2.90)	0.887	0.69	(0.23–2.12)	0.519
SL only	8.4	1.52	(0.59–3.94)	0.387	1.29	(0.43–3.90)	0.647	1.54	(0.53–4.46)	0.425
PA + ST	8.2	0.84	(0.28–2.54)	0.761	0.42	(0.09–1.95)	0.267	0.42	(0.09–1.99)	0.275
PA + SL	9.3	1.16	(0.45–3.00)	0.755	0.73	(0.22–2.38)	0.600	0.54	(0.15–2.05)	0.368
ST + SL	5.3	1.13	(0.34–3.78)	0.837	0.32	(0.04–2.58)	0.285	0.34	(0.04–2.75)	0.312
PA + ST + SL	6.5	0.34	(0.07–1.56)	0.163	0.25	(0.03–1.97)	0.187	0.26	(0.03–2.07)	0.202

BMI – body mass index, CI – confidence interval, FM% – fat mass percentage, OR – odds ratio, PA – physical activity, Ref – reference group, SD – standard deviation, ST – screen time, SL – sleep, VAT – visceral adiposity tissue

The model was adjusted for sex, age, birth weight, maternal obesity, and unhealthy diet

Children with ≥60 min/day of moderate-to-vigorous physical activity, < 2 h/day of recreational ST, and uninterrupted sleep for 9–11 h/day were considered to meet the overall 24-hour movement guidelines

**Table 3:** Odds of excess adiposity in adolescents who met different numbers and specific combinations of recommendations within the 24-hour movement guidelines (*n* = 324)

	Adherence	BMI ***z***-score > 1 SD			>85th percentile of FM%			>85th percentile of VAT		
	%	OR	(95% CI)	***p***-value	OR	(95% CI)	***p***-value	OR	(95% CI)	***p***-value
**Number of recommendations**										
None	36.4	Ref.			Ref.			Ref.		
One	47.2	0.51	(0.27–0.96)	0.042	0.49	(0.23–1.03)	0.059	0.39	(0.19–0.81)	0.012
Two	14.2	0.33	(0.11–0.94)	0.039	0.69	(0.25–1.88)	0.468	0.25	(0.07–0.90)	0.034
Three	2.2	1.27	(0.23–7.16)	0.787	0.77	(0.09–6.95)	0.813	0.25	(0.07–5.88)	0.704
**Combinations of recommendations**										
Did not meet any recommendation	36.4	Ref.			Ref.			Ref.		
PA only	6.8	0.67	(0.20–2.22)	0.510	0.23	(0.03–1.79)	0.158	0.18	(0.02–1.39)	0.100
ST only	26.2	0.38	(0.17–0.89)	0.025	0.34	(0.13–0.93)	0.035	0.27	(0.10–0.74)	0.010
SL only	14.2	0.63	(0.26–1.57)	0.325	0.90	(0.34–2.34)	0.824	0,72	(0.28–1.86)	0.500
PA + ST	4.9	0.34	(0.07–1.65)	0.179	0.59	(0.12–2.88)	0.514	0.22	(0.03–1.79)	0.157
PA + SL	2.5	1.56	(0.27–8.85)	0.618	2.15	(0.38–12.23)	0.387	1.79	(0.32–10.10)	0.510
ST + SL	6.8	0.11	(0.01–0.89)	0.039	0.42	(0.09–2.01)	0.281	NA		
PA + ST + SL	2.2	1.20	(0.21–6.79)	0.836	0.72	(0.08–6.58)	0.774	0.62	(0.07–5.59)	0.668

BMI – body mass index, CI – confidence interval, FM% – fat mass percentage, OR – odds ratio, PA – physical activity, Ref – reference group, SD – standard deviation, ST – screen time, SL – sleep, VAT – visceral adiposity tissue

NA, not applicable – indicating insufficient sample size for estimation

The model was adjusted for sex, age, birth weight, maternal obesity, and unhealthy diet

Adolescents with ≥60 min/day of moderate-to-vigorous physical activity, < 2 h/day of recreational ST, and uninterrupted sleep for 8–10 h/day were considered to meet the overall 24-hour movement guidelines

The results presented in Tables 2 and 3 show that adolescents who met the ST only recommendation had significantly lower odds of having excess adiposity, which was defined by BMI *z*-score (OR = 0.38, 95% CI: 0.17–0.89), FM% (OR = 0.34, 95% CI: 0.13–0.93), and VAT (OR = 0.27, 95% CI: 0.10–0.74) compared with those who did not meet any recommendation. Moreover, significantly lower odds of having a high BMI *z*-score (OR = 0.11, 95% CI: 0.01–0.89) was observed in adolescents when the combination of ST and sleep recommendations was met. No significant associations were found in children.

## Discussion

This study revealed that a low proportion of children and adolescents met all three recommendations within the 24-hour movement guidelines. Meeting one recommendation or any two recommendations within the 24-hour movement guidelines was associated with a reduced risk of excess adiposity in adolescents. Specifically, ORs for excess adiposity were significantly lower when adolescents met the ST only recommendation and the combination of the ST and sleep recommendations. No significant associations were found in children.

Surprisingly, the present study showed no associations between meeting all three recommendations within the 24-hour movement guidelines and adiposity indicators. This finding is in contrast to other published studies reporting that a favorable adiposity status is associated with meeting more recommendations [21, 22]. The same trend was also found for several cardiometabolic markers and physical fitness [38]. One possible explanation for such finding may be the very low adherence to all three recommendations in our sample. Therefore, the results of the analysis should be interpreted with caution because the used regression models rely on asymptotics.

The results of the present study suggest that reducing the time spent in front of a screen could be a potential target for preventing childhood obesity. Our findings are in accordance with previously published studies reporting that meeting the ST recommendation is associated with lower odds of obesity whether or not PA and sleep recommendations are met [22, 39]. It can be assumed that ST, a main contributor to the total SB, accumulates with a few prolonged uninterrupted sedentary periods, which are associated with excess adiposity independently of PA [40].

Our study extends the existing literature by showing that participants who met the combination of ST and sleep recommendations had lower odds of having a high BMI *z*-score. This finding could be attributable to the compensatory change between 24-hour movement behaviors that are typical examples of compositional data [41]. We can therefore hypothesize that a decrease in the time spent in front of a screen leads to an increase in the remaining behaviors within the 24-h period, especially to an increase in sleep duration. The study by Lin et al. [42] supported this assumption by showing that a lower amount of time spent in SB is associated with a higher sleep duration in the subsequent night. This relationship seems to be bidirectional, as an association between sleep duration and SB in the following day was also confirmed. Thus, intervention programs targeting ST reduction and improving sleep duration are warranted to reduce the increasing prevalence of childhood obesity.

Another original finding was that our analysis confirmed associations only in adolescents but not in children. This finding could be explained by a decrease in family influence (i.e., parental modeling and encouragement, household rules, and restrictions) with respect to health behaviors during the transition from childhood to adolescence [43]. It can be assumed that adolescents are less restricted by parents in terms of ST than children. Adolescents also have higher bedtime autonomy, which is associated with a short sleep duration [44]. Moreover, this relationship is moderated by overuse of electronic devices before going to sleep. In addition to the fact that extensive ST and lack of sleep are significant contributors to childhood obesity, such behaviors are also related to other obesogenic behaviors, such as unhealthy eating [45]. For this reason, adolescents who meet the ST and sleep recommendations are probably less exposed to other obesogenic behaviors than children, and therefore have higher odds of having a favorable adiposity status. Future research should verify this hypothesis, as this is beyond the scope of the present study.

The major strength of the present study was using the 24-h multi-day raw accelerometer data in assessing PA and sleep. Another strength is that the research sample was not limited to a narrow age group, as in previous studies [22]. Finally, the inclusion of demographic and behavioral measures allowed us to adjust the regression models for several confounding factors.

This study had some limitations. First, the design was cross-sectional, which limits causal inferences between the adherence to the 24-hour movement guidelines and the adiposity status. Second, although the study participants were recruited from Czech cities of different sizes, the study sample was not fully representative because it mainly represents the urban population. Thus, the potential of our findings to be generalized to the rural population may be limited. Third, the estimated amount of time spent in MVPA may be biased in adolescents as the cut-off point (i.e., 201 m*g*) was proposed for children. Fourth, the determination of self- or parental-reported ST may be difficult, as multi-tasking has become a common phenomenon in adolescents [46] and this behavior is typically underreported [47]. Finally, considering that whole-body bioimpedance analysis is not considered a gold standard method for assessing VAT, the results of this analysis could be biased [48].

## Conclusions

The present study showed no associations between meeting all three recommendations within the 24-hour movement guidelines and adiposity indicators. However, meeting the ST only recommendation or the combination of the ST and sleep recommendations was associated with a favorable adiposity status. Therefore, the combined effect of ST and sleep should be considered in establishing effective intervention strategies that target the prevention of childhood obesity.

## Data Availability

The dataset analyzed during the current study is available in the Figshare repository, 10.6084/m9.figshare.12017886.

## Abbreviations

PA: Physical activity
MVPA: Moderate-to-vigorous physical activity
SB: Sedentary behavior
ST: Recreational screen time
BMI: Body mass index
FM%: Fat mass percentage
VAT: Visceral adipose tissue
SD: Standard deviation
OR: Odds radio
CI: Confidence interval
